# Enhanced collagen type I synthesis by human tenocytes subjected to periodic in vitro mechanical stimulation

**DOI:** 10.1186/1471-2474-15-386

**Published:** 2014-11-21

**Authors:** Elise Huisman, Alex Lu, Robert G McCormack, Alex Scott

**Affiliations:** Department of Physical Therapy, University of British Columbia, Vancouver, Canada; Centre for Hip Health and Mobility, Vancouver Coastal Health and Research Institute, Vancouver, Canada; Department of Orthopaedic Surgery, University of British Columbia, Vancouver, Canada

## Abstract

**Background:**

Mechanical stimulation (e.g. slow heavy loading) has proven beneficial in the rehabilitation of chronic tendinopathy, however the optimal parameters of stimulation have not been experimentally determined. In this study of mechanically stimulated human tenocytes, the influence of rest insertion and cycle number on (1) the protein and mRNA levels of type I and III collagen; (2) the mRNA levels of transforming growth factor beta (*TGFB1*) and scleraxis (*SCXA*); and (3) tenocyte morphology, were assessed.

**Methods:**

Human hamstring tenocytes were mechanically stimulated using a Flexcell® system. The stimulation regimens were 1) continuous and 2) rest-inserted cyclic equiaxial strain at a frequency of 0.1 Hz for 100 or 1000 cycles. Data were normalized to unstimulated (non-stretched) control groups for every experimental condition. qPCR was performed to determine relative mRNA levels and quantitative immunocytochemistry image analysis was used to assess protein levels and cell morphology.

**Results:**

Collagen type I mRNA level and pro-collagen protein levels were higher in tenocytes that were subjected to rest-inserted mechanical stimulation, compared to continuous stretching (p < 0.05). Rest insertion and increased cycle number also had significant positive effects on the levels of mRNA for *TGFB1* and *SCXA* (p < 0.05). There was no direct relation between cell morphology and gene expression, however mechanical stimulation, overall, induced a metabolically active tenocyte phenotype as evidenced by cells that on average demonstrated a decreased major-minor axis ratio (p < 0.05) with greater branching (p < 0.01).

**Conclusions:**

The incorporation of rest periods in a mechanical stretching regimen results in greater collagen type I synthesis. This knowledge may be beneficial in refining rehabilitation protocols for tendon injury.

**Electronic supplementary material:**

The online version of this article (doi:10.1186/1471-2474-15-386) contains supplementary material, which is available to authorized users.

## Background

Tendons experience varying loads in their natural environment. Excessive mechanical loading [1] on the one hand, or stress deprivation [2, 3] on the other, can lead to tendon damage or degeneration. Conversely, physiological mechanical stimulation promotes ongoing collagen synthesis and repair activity by resident tendon fibroblasts (tenocytes) [4]. The physiological variables (strain level, frequency, repetition number, etc.) which determine the tissue and cellular responses of tendons to mechanical stretching have been studied to some extent [5], but the optimal conditions to prevent injury or to promote collagen synthesis and repair are incompletely understood.

Increased knowledge of the parameters of mechanical stretching which promote collagen synthesis by tenocytes could help to refine exercise prescription, especially when considering that mechanical stimulation of tendons through exercise is a cornerstone of rehabilitation following acute or chronic tendon injury [6]. An exercise regimen (3 sets of 15 heel drops performed slowly, twice per day, with weight gradually increased to patient tolerance) has been shown to be effective for chronic Achilles tendinopathy in both the short and long term [6]. One proposed mechanism of action of this type of exercise regimen is a stimulation of collagen type I production by tenocytes which, in the long term, could lead to an increased tendon strength and modulus [6].

Experimental data have suggested that there may be a window of appropriate mechanical stimulation defined by variables such as repetition number and strain magnitude. A strain magnitude dependency was reported for mRNA and protein levels of collagen type I [7], whereas an inverse strain magnitude dependency was found for *Mmp1*[8] (a metalloproteinase with collagenase activity). Several studies have shown that tenocytes undergoing mechanical stretching increase their expression of the genes for collagen type I and III as well regulatory genes and growth factors which influence collagen synthesis such as *TGFB1* and *SCXA*[4, 8–10].

Surprisingly, an equivalent magnitude of increase in collagen type I synthesis rate in the human patellar tendon was observed following both long distance running (36km) and strength training (10 sets of 10 maximal knee extensions) [11, 12]. This finding has led to the speculation that collagen type I production by tenocytes might plateau after a number of loading repetitions as low as n = 100 [11]. Desensitization to continuous load cycles is a phenomenon known to occur in bone cells [13]. More importantly, the insertion of recovery periods during the mechanical stimulation restored the mechanosensitivity in bone cells [13]. The insertion of rest periods during cyclic stretching experiments performed on adolescent and aged murine tibia resulted in enhanced bone formation compared with controls. The level of bone formation observed in response to rest-inserted loading was similar to a loading regimen that doubled the load and cycle number [14, 15]. This may indicate that a high load and cycle number are not necessary to elicit a maximal response if rest periods are included. A study of osteogenesis in mice tibia showed that rest periods inserted in between short bouts of stretching had a larger effect on osteogenesis than continuous stretching. While several studies have displayed the positive effects of rest insertion on bone formation [13–17], the effect of rest-insertion on the load-induced expression of collagen genes in tenocytes has yet to be examined.

In vivo, tenocytes typically display an elongated morphology, oriented along the longitudinal direction of the tendon [18], while in response to increased mechanical loading, they may adopt a more rounded morphology with more prominent cytoplasm. *In vitro*, it has been shown that gene expression and protein production are influenced by cell attachment and spreading [19]. Li et al. reported that more elongated human tendon fibroblasts expressed higher collagen type I levels compared to less elongated cells, while the cell spreading area was constant [20]. Alternately, other studies have shown that tendon fibroblasts may adopt a more rounded shape when more metabolically active. In the human patellar tendon, ovoid tendon cells expressed higher levels of *TGFB1* and pro-collagen type I compared to elongated tenocytes [21]. To our knowledge, the relation of tendon cell shape and collagen gene expression has not been directly investigated in mechanically stimulated tenocytes.

Tenocytes were mechanically stimulated to test the hypothesis that rest insertion compared to continuous stretching initially up-regulates *SCXA* and *TGFB1* mRNA levels [22–24] and is accompanied by enhancement of gene expression and protein levels of pro-collagen type I and III, whereas low (100) and high (1000) cycle numbers would have equivalent effects on these same variables. As a secondary question, the influence of mechanical stimulation on cell morphological features was analyzed.

## Methods

### Cell culture

Human hamstring tendon pieces were obtained from ACL reconstruction surgery, after informed consent was obtained. Ethics approval was obtained from the University of British Columbia. Tendon tissue was trimmed to remove fat and muscle then washed with phosphate buffered saline (PBS) and enzymatically digested with filtered 1.5 mg/ml collagenase (Clostridopeptidase A; Sigma, Oakville, Ontario, Canada) in serum-free Dulbecco’s Modified Eagle Medium (DMEM; HyClone, South Logan, Utah, USA) for 30 minutes at 37°C with shaking; for 5 additional minutes, trypsin (1× TrypLE™ Select, Gibco, Life Technologies, Burlington, ON, Canada) was added. The mixture was centrifuged at 1,200 rpm for 5 minutes and the cell pellet was resuspended. Tenocytes were cultured using Hyclone DMEM/high glucose media supplemented with 10% fetal bovine serum and 1% penicillin/streptomycin (Thermo Scientific, Ottawa, ON, Canada).

### Mechanical stimulation

Cells were passaged at a concentration of 32,500 cells/ml. 65,000 cells per well were plated onto 6-well BioFlex® - collagen type I coated culture plates (Flexcell International Corp., Hillsborough, NC, USA). After 48 hours, the cells were subjected to mechanical stimulation using a sinusoidal waveform at a frequency of 0.1 Hz and a strain of 10%. The frequency of 0.1 Hz was based on findings of Kongsgaard et al. who demonstrated that slow (e.g. 6-8 second repetition duration), heavy resistance exercise was beneficial in the rehabilitation of patellar tendinopathy resulting in enhanced collagen synthesis [25]. The equiaxial strain of 10% applied to the BioFlex® plates has been previously reported to result in an average strain experienced by the cells of approximately 3-5% [26]. The study consisted of four experimental groups with a combination of low or high cycle number and with or without rest. The precise groups were 1) 100 cycles of continuous stretching, 2) 100 cycles with 10s rest after every cycle, 3) 1000 cycles of continuous stretching, 4) 1000 cycles with 10 s rest after each cycle. For every experiment group there was a corresponding unstimulated control group, whose cells were otherwise treated identically and harvested at the same time points. All experiments were performed in biological and technical triplicates.

### RNA extraction and quantitative polymer chain reaction (qPCR)

Cells were lysed using lysis buffer (Thermo Scientific, Ottawa, ON, Canada) with 1% β mercaptoethanol and stored at -80°C until further processing. Ribonucleic acid (RNA) was extracted according to the manufacturers’ instructions using the GeneJet RNA purification kit (Thermo Scientific, Ottawa, ON, Canada) and stored at -80°C until further processing. Complementary deoxyribonucleic acid (cDNA) was synthesized using 100mM dNTP set (Life Technologies, Burlington, ON, USA) and stored at -20°C. qPCR was performed in triplicate using SYBR Green (FastStart Universal SYBR Green Master ROX, Roche Diagnostics Corporation, Indianapolis, IN, US) on a 7500 Fast Real – Time PCR System (Applied Biosystems, Life Technologies, Burlington, ON, USA). The primers used (Table 1) were designed for the target human genes. Values for mRNA transcript levels were normalized to corresponding Glyceraldehyde 3-phosphate dehydrogenase (*GAPDH*) values.

**Table 1:** **RT-qPCR primers**

Target	Forward Primer	Reverse Primer
GAPDH	TCTTTTGCGTCGCCAGCCGAG	TGACCAGGCGCCCAATACGAC
Collagen type I	TGTTCAGCTTTGTGGACCTCCG	CGCAGGTGATTGGTGGGATGTCT
Collagen type III	AATCAGGTAGACCCGGACGA	TTCGTCCATCGAAGCCTCTG
TGFB	GCAACAATTCCTGGCGATACC	AAAGCCCTCAATTTCCCCTCC
Scleraxis A	AGAACACCCAGCCCAAACA	TCGCGGTCCTTGCTCAACTT

### Immunocytochemistry for pro-collagen types I and III

Cells of all experimental groups and corresponding controls were fixed on the BioFlex plate stretchable substrate in 4% paraformaldehyde (w/v) for 15 minutes at room temperature and stored at 4°C until further processing. Substrate sections of 1×1cm were cut from similar locations of the membrane using a scalpel and mounted on glass slides, and permeabilized in 0.4% Triton X-100 for 5 min after which the sections were blocked with PBS +1% BSA +0.2% Tween20 solution for 30 minutes. A mixture of primary antibodies against both pro-collagen type I (Developmental Studies Hybridoma Bank. Iowa City, IA, US) and pro-collagen type III (Acris Antibodies, Inc. San Diego, CA, US) was applied at a concentration of 1:500 for 1 hour in the dark. This was followed by the incubation with the secondary antibody solution (Alexa fluor 488 goat anti-rabbit and 596 goat anti-mouse, Life Technologies, Burlington, ON, US) at a concentration of 1:400 for 30 min in the dark. Finally, nuclei were stained using Hoechst (33342 Thermo Scientific, Rockford, IL, USA) antibody in a dilution of 1:10,000 PBS for 2 minutes. All staining procedures were performed in a humidified chamber at room temperature.

### Image acquisition

Fluorescent micrographs were obtained using a Zeiss Axio Observer.A1 (Zeiss, Oberkochen, Germany) with a 10x objective. For each field of view, three images were taken on each separate fluorescent channel to represent nuclei and pro-collagens types I and III. Nuclei were identified by thresholding using CellProfiler™ software; the pro-collagen type I and III fluorescent stains associated with each nuclei were then identified. Three images per fluorescent channel and per field of view were obtained, resulting in data from approximately 300 tendon cells of each experimental and control group.

### Measurement of pro-collagen I and III protein

Protein levels of pro-collagen type I and III were operationally defined as the intensities of the fluorescent channel of pro-collagen type I and III, respectively, as determined using AxioVision software (Zeiss, Oberkochen, Germany). The fluorescence intensities were obtained through the identified pro-collagen I and III labeling described in the previous paragraph of the methodology. For each individual fluorescent channel, the pixel intensities on a scale from 0 to 1 within the identified area were averaged to obtain mean intensity.

### Analysis of cell morphology

Cell morphology values were obtained from each cell demonstrating positive pro-collagen type III fluorescent labeling. This pro-collagen type III based method was chosen because all tendon cells examined constitutively demonstrated robust labeling (of varying intensity, as described above) throughout their cytoplasm, allowing the overall cell morphology to be clearly assessed.

To obtain solidity values a convex hull was drawn around the cell surface. The solidity was derived as the ratio of surface area of the stain to total surface area of the convex hull, thus representing an approximation of the extent of cell branching. A perfectly smooth object would therefore be assigned a value of 1, whereas the more branched the cell, the lower the solidity value.

The ratio between major and minor axis per cell, was also obtained from the elliptical outline around the pro-collagen type III immunolabeling as an additional indication of cell shape. An ellipse was fitted around the cell and the length (in pixels) of the major and minor axis of the ellipse. The resultant output images were used to manually filter the datasets by deleting erroneously identified object sets caused by image artefacts or overlapping cells.

### Statistics

Linear mixed model analysis was performed using SPSS (SPSS Inc., Chicago, IL, USA) to test for statistically significant differences in mRNA levels. Repeated Generalized Estimating Equations within Generalized Linear Models using SPSS was used to determine protein level, cell solidity and ratio of major/minor axis among the groups. The model tested for main effects of mechanical stimulation vs controls, rest inserted vs continuous stretching, and low (100) vs high (1000) cycle number. The mRNA data were expressed as relative quantity (RQ, relative to unstimulated tenocytes harvested under identical conditions at the same time) after normalizing the raw data to the housekeeping gene *GAPDH*. RQ values were then log transformed before statistical analysis to obtain normal distributions. A *p* value of ≤0.05 was considered statistically significant for all statistical tests. The qPCR results are reported as the mean difference between low and high cycle number and continuous and rest inserted mechanical stimulation groups with a 95% confidence interval (CI) of the difference. The protein and cell morphology results were reported as mean and 95% CI. For both methods CI adjustments were made using Sidak correction. The figures depict the main effects tested by the linear models, with each variable (continuous vs rest-inserted loading, 100 vs 1000 repetitions) depicted in a separate figure.

## Results

### Mechanical stimulation of human tendon cells compared to un-stimulated cells

The cyclic stretching regimens were well tolerated by the tenocytes (i.e. no evidence of cell death and no difference in RNA concentrations between stimulated and unstimulated groups). Overall, mechanical stimulation led to significantly greater collagen type I and *SCXA* mRNA levels (p < 0.05) compared with unstimulated cultures (8 h, Table 2).

**Table 2:** **The effect of mechanical stimulation on mRNA expression compared with unstimulated controls**

Group	Collagen type I	Collagen type III	Scleraxis-A	Transforming growth factorβ
**Rest**	1.15(1.00-1.34)*	0.81(0.59-1.11)	2.62(2.09-3.27)ƚ	1.21(0.83-1.76)
**Continuous**	0.86(0.70-1.05)	0.67(0.46-0.98)*	1.79(1.22-2.64)ƚ	0.80(0.56-1.16)
**100 cycles**	0.90(0.73-1.10)	0.93(0.60-1.55)	3.33(2.93-3.79)ƚ	1.20(0.89-1.63)
**1000 cycles**	1.20(1.03-1.40)*	0.66(0.49-0.89)ƚ	1.43(1.03-1.99)*	0.83(0.50-1.38)

The data is displayed as the back transformed mean difference of experimental group minus control group (confidence interval of difference). Values > 1 depict larger values for the experimental group compared to the unstimulated control cultures. The *indicates a significant difference of p < 0.05, the ƚ indicates significant difference of p < 0.01. The back transformed means (CI) are not equivalent to means of the original variable due to mathematical twisting [27].

The immunohistochemistry also indicated that the pro-collagen type I intensity of the stretched group was increased after 24 h (p < 0.01) and had a mean of 0.205(0.203-0.207) while the control group displayed a mean of 0.176(0.174-0.179). The stretched group showed a higher (p < 0.01) mean of 0.262(0.261-0.264) for pro-collagen type III intensity while the control group had value of 0.238(0.235-0.240).

### Rest-inserted vs continuous stretching

**Figure 1:**
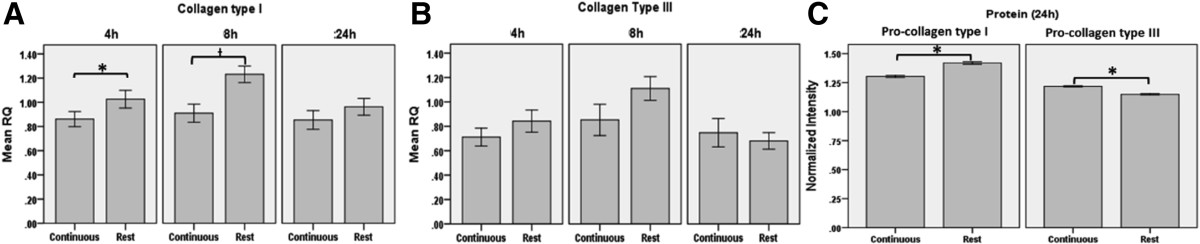
**Effect of rest-inserted stretching vs continuous stretching on mRNA expression of collagen type I (A) and III (B) and protein levels of pro-collagen I and III (C).** The cells that underwent rest inserted stretching showed an increased mRNA expression of collagen type I **(** **A** **)** at 8 h post stretching (p < 0.05) compared to continuously loaded cells. Collagen type III mRNA expression did not significantly change **(B)**. The pro-collagen type I was significantly greater (p < 0.05) in the rest insertion group compared to the continuously loaded, while the continuous group had an increased (p < 0.05) pro-collagen type III value at 24 hours post stretching **(** **C** **)**. The *indicates a significant difference of p < 0.05, the ƚ indicates significant difference of p < 0.01, the error bars indicate the standard error. All data points from each group were normalized to unstretched controls harvested at every time point.

Rest insertion resulted in a greater protein level of pro-collagen type I [1.421(1.399-1.442)] (24 h, p < 0.01) compared to continuous stretching [1.303(1.284-1.322)] while pro-collagen type III levels were greater with continuous stretching [1.223(1.211-1.235)] compared with the cells stretched in the rest-inserted regimen [(1.142(1.130-1.153), 24 h, Figure 1, p < 0.01]. Overall, periods of rest insertion had a positive effect on mRNA levels for collagen type I with a mean difference (CI of difference) of 1.35(1.10-1.65) *SCXA* 1.30(1.02-1.65) and *TGFB1* [1.70(1.10-2.65), 8 h, p < 0.05]. Collagen type I mRNA levels 1.20(1.01-1.43) were increased as early as 4 h (p < 0.05).

**Figure 2:**
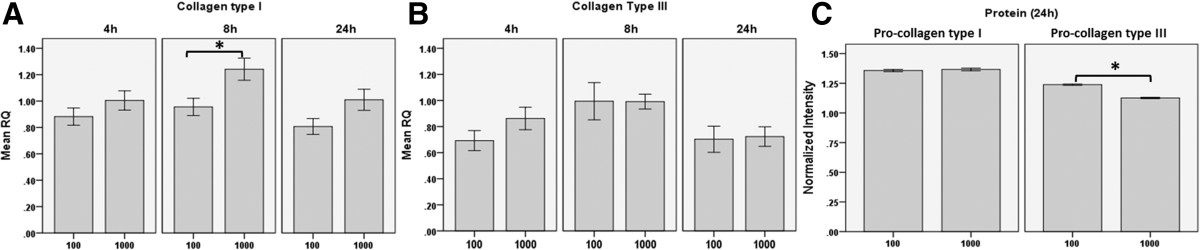
**Effect of cycle number on mRNA expression and protein level of collagen type I and III.** The high cycle number (1000) caused elevated Collagen type I mRNA levels (p < 0.05). Pro-collagen type III protein levels **(C)** were upregulated in the 100 cycles regimen (p < 0.05, 24 h). The *indicates a significant difference of p < 0.05, the indicates **ƚ** significant difference of p < 0.01, the error bars indicate the standard error. All data points from each group were normalized to unstretched controls harvested at every time point.

### Low vs high cycle number

The pro-collagen type III protein level was greater (p < 0.01) in the 100 cycles stretch regimen [1.243(1.231-1.254)] compared to the 1000 cycle group [1.122(1.111-1.134), 24 h, Figure 2].

The cells stimulated for 1000 cycles showed higher expression of *TGFB1* 1.49(1.20-1.85), 4 h, p < 0.01], and collagen type I [1.31(1.07-1.60, 8 h, p < 0.05] compared to the 100 cycle regimen.

### Cell morphology

**Figure 3:**
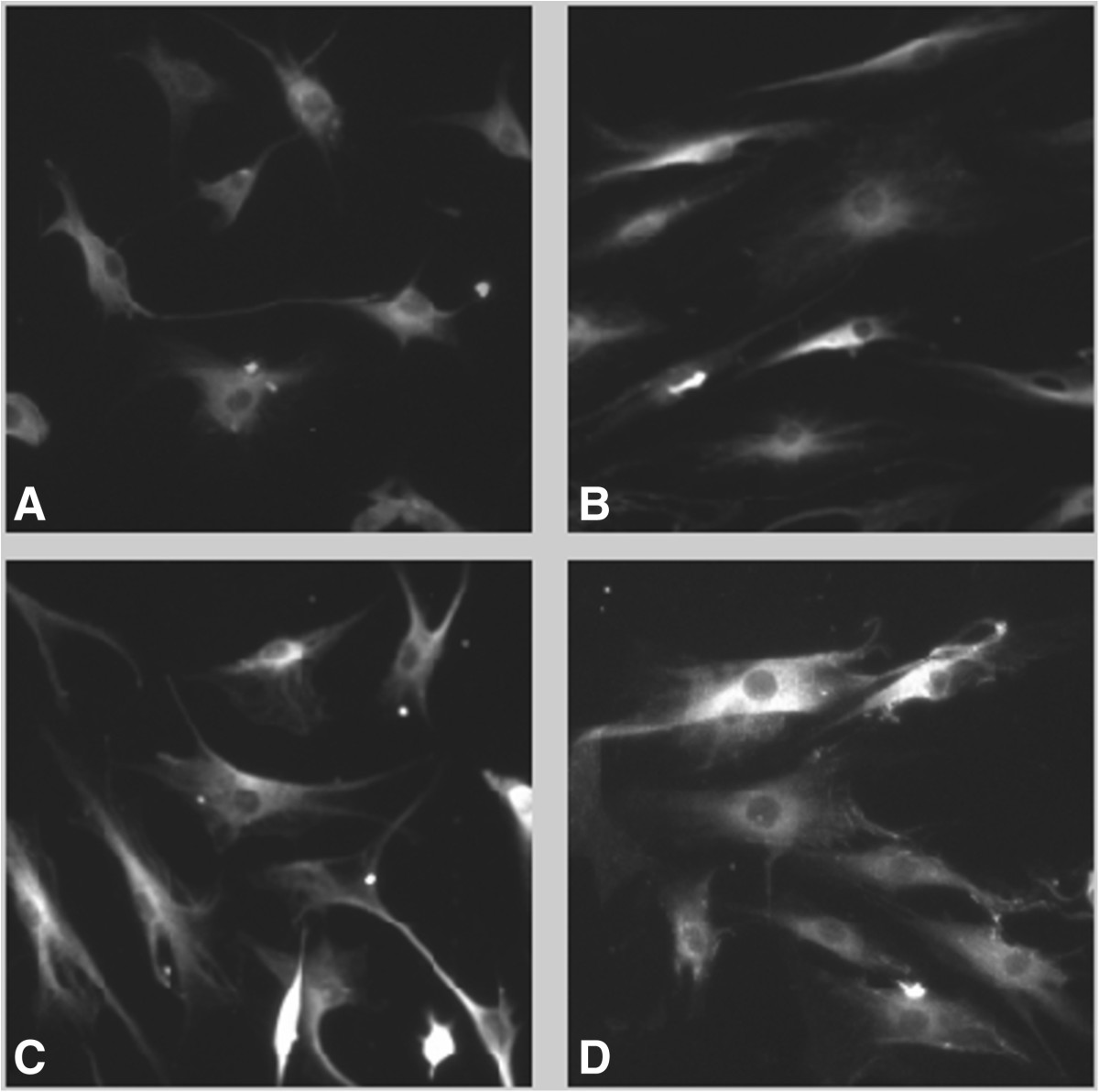
**Immunocytochemistry images (pro-collagen type III), 10× magnification. A)** Un-stimulated control cells, **B)** 100 cycles continuously stretched cells, **C)** 1000 continuously stretched cells and **D)** 1000 cycles rest insertion cells. Mechanically stimulated cells (especially **C** and **D**) appear more spread out, with a correspondingly reduced major/minor axis.

The solidity of tendon cells was greater when subjected to mechanical stimulation [0.621(0.618-0.624)] compared with unstimulated controls [0.601(0.597-0.605)] (p < 0.01), indicating increased cell branching with mechanical stimulation. The major/minor axis, a measure of cell shape, in mechanically stretched tenocytes was lower [2.845(2.815-2.875)] than the non-stretched control group (p < 0.01, 3.005(2.958-3.053), Figure 3) indicating increased cell rounding with mechanical stimulation.The major to minor axis ratio was lower (24 h, p < 0.05, Figure 3) with the 1000 cycles stretching regimen [2.159(1.757-2.561)] compared to the 100 cycle regimen [2.778(2.748-2.808)].

## Discussion

The main finding of this study is that the insertion of rest periods between cycles of mechanical stimulation of tenocytes resulted in greater expression of collagen type I mRNA and protein compared with continuously stimulated tenocytes.

Mechanical stimulation of cells results in mechanotransduction; the transmission and conversion of a mechanical stimulus into a biological response, through a variety of mechanisms. There are three main stages of mechanotransduction: mechanocoupling, cell-to-cell communication, and effector cell response. Mechanocoupling is the process whereby the applied load is transmitted through the tissues and cells, resulting in different types of cellular deformation (strain, compression, fluid flow and shear [28]), and this deformation is translated into a biochemical response which can include the opening of ion channels such as stretch-activated calcium channels, and the activation of transmembrane signaling proteins such as integrins and G-protein coupled receptors [29]. The mechanically stimulated cell may also spread the signal to adjacent cells (cell-to-cell communication, involving the passage of calcium ions via gap junction, for instance) thereby amplifying the response [30]. In response to elevated intracellular calcium and other signals, enzyme activity is initiated within the cell (e.g. activation of MAPK family members) leading to gene transcription and production of protein which can include newly synthesized extracellular matrix, or autocrine/paracrine substances like TGFB or IGF-I which further amplify the adaptive responses [22]. Most of these processes have been documented to some extent in tendon cells, which are known to be highly mechanoresponsive. The adaptive response of tenocytes has been shown, *in vivo*, to be related to the magnitude of applied strain; e.g., exercise in a shortened (low strain) position leads to less tendon adaptation than exercise in a lengthened (high strain) position [31].

LaMothe & Zernicke [16] and Hanson et al. [17] researched the effect of rest insertion and showed that rest insertion incorporated in loading protocols had a larger effect on mineral deposition and osteogenesis than loading only [16, 17]. Commonly, mechanical stimulation is applied as a continuous bout of stretch at a certain frequency [32]. The current results suggest that rest incorporation stimulates the expression of collagen type I and collagen type III mRNA to a greater degree than continuous stimulation.

The number of repetitions of applied mechanical stretching can elicit different gene expression patterns. In this study, at a frequency of 0.1 Hz the high cycle number (1000) increased the *TGFB1* (4 h) and collagen type I (8 h) mRNA expression, while no change in collagen type III was observed. In a human *in vitro* patellar tendon model Bosch et al. showed an increase in amino terminal pro-collagen type III propeptide (P-III-NP) after 1800 and 3600 stretch cycles at a strain of 5% and a frequency of 1 Hz. The higher cycle number (3600) resulted in a higher P-III-NP elevation (32%) compared to the lower cycle number (1800) which demonstrated a 21% increase. The carboxyterminal pro-collagen type I propeptide levels demonstrated a 50% increase after 3600 cycles of stretching, but not 1800. These findings are in line with our results; a higher cycle number leads to a more increased collagen type I mRNA expression.

*SCXA* regulates the transcription of collagen type 1a1 and TGFB is known to induce collagen type I expression [22, 23, 33]. In the current study the cells stretched on a regimen of 1000 cycles upregulated *SCXA* (4 h), *TGFB1* (4 h) and collagen type I (8 h) mRNA expression; this pattern is in line with expectations of upregulated *SCXA* and *TGFB1* preceding elevated levels of collagen type I and collagen type III are present post stimulation.

It has been shown that mechanical stresses applied to the cells lead to altered forces within the cell which play important roles in the control of cell shape and cell function [34, 35]. For example, the application of shear stress e.g. caused by blood flow, to endothelial cells resulted in altered cell shape from cobblestone to aligned in the direction of the flow and the formation of actin stress fibers [36]. The results indicate that mechanical stimulation may induce a more metabolically active tenocyte phenotype, with tendon cells that are less elongated, and more branched, particularly when subjected to cyclic stretching that incorporates rest insertion and longer durations; these same regimens are associated with higher expression levels of a tenocyte transcriptional regulator (*SCXA*) and a key tendon extracellular matrix protein (type 1 collagen). These findings are in contrast with those of Li et al., who found that more elongated cells had a higher collagen type I protein expression [20], however they are in keeping with those of Chuen et al. who found less elongated tenocytes to express higher levels of type I collagen [21]. Clearly, cell shape is dynamically regulated by mechanical stimulation and a clear relation with gene expression is not to be expected. It may also be worth pointing out that older, morphological categorizations of tendon cells based on their shape (e.g. “tenoblasts” being a more rounded subpopulation of tendon cells compared to the elongated tenocytes) may not be valid [37], and that tenocyte rounding, sometimes taken to be a feature of tendinosis [38], may in fact be associated with an adaptive response and should not necessarily be interpreted as pathological.

A limitation of this study was that the observed changes in mRNA expression were of small magnitude; however, they were in keeping with a significant upregulation at the protein level and with the knowledge that tendon is a relatively slow-adapting tissue. Furthermore, our cell shape observations, although based on automated measurements of many cells, do not account for possible changes in cell thickness. There is also an inherent limitation of *in vitro* studies; the response to stretching may also have been more accentuated if the tendon cells were located within their native extracellular matrix, rather than cultured two dimensionally – a condition which likely disturbs integrin-mediated signaling which is thought to contribute to mechanoresponsiveness [39]. Nonetheless, *in vitro* studies with tenocytes in two dimensional culture have replicated many of the mechanically-induced responses that are known to occur *in vivo* and may therefore continue to serve as a useful experimental system.

## Conclusion

In this study, we demonstrated that periods of rest insertion are beneficial for collagen synthesis by human tenocytes. One implication of these findings is that when using exercise as a rehabilitative measure for people with chronic tendinopathy, allowing an adequate time to recovery after every stretch cycle may induce a more substantial adaptive response. Further studies could investigate the role of mechanotransduction pathways in response to rest-inserted stretching (e.g. the role of calcium signaling) and to examine whether a rest-inserted exercise program (e.g. 10 s rest after every repetition) results in improved tendon adaptation in humans. In addition, further optimization of mechanical properties capable of stimulating collagen synthesis could be undertaken, including strain rate and frequency.

## Authors’ original submitted files for images

Below are the links to the authors’ original submitted files for images. Authors’ original file for figure 1 Authors’ original file for figure 2 Authors’ original file for figure 3 Authors’ original file for figure 4 Authors’ original file for figure 5 Authors’ original file for figure 6 Authors’ original file for figure 7

## References

[1] Scott A, Khan KM, Heer J, Cook JL, Lian O, Duronio V (2005). High strain mechanical loading rapidly induces tendon apoptosis: an ex vivo rat tibialis anterior model. Br J Sports Med.

[2] Thornton GM, Shao X, Chung M, Sciore P, Boorman RS, Hart D a, Lo IKY (2008). Changes in mechanical loading lead to tendonspecific alterations in MMP and TIMP expression: influence of stress deprivation and intermittent cyclic hydrostatic compression on rat supraspinatus and Achilles tendons. Br J Sports Med.

[3] Gardner K, Arnoczky SP, Caballero O, Lavagnino M (2008). The effect of stress-deprivation and cyclic loading on the TIMP/MMP ratio in tendon cells: an in vitro experimental study. Disabil Rehabil.

[4] Heinemeier KM, Olesen JL, Haddad F, Langberg H, Kjaer M, Baldwin KM, Schjerling P (2007). Expression of collagen and related growth factors in rat tendon and skeletal muscle in response to specific contraction types. J Physiol.

[5] Lavagnino M, Arnoczky SP (2005). In vitro alterations in cytoskeletal tensional homeostasis control gene expression in tendon cells. J Orthop Res.

[6] Alfredson H, Cook J (2007). A treatment algorithm for managing Achilles tendinopathy: new treatment options. Br J Sports Med.

[7] Jiang C, Shao L, Wang Q, Dong Y (2012). Repetitive mechanical stretching modulates transforming growth factor-β induced collagen synthesis and apoptosis in human patellar tendon fibroblasts. Biochem Cell Biol.

[8] Lavagnino M, Arnoczky SP, Tian T, Vaupel Z (2003). Effect of Amplitude and Frequency of Cyclic Tensile Strain on the Inhibition of MMP-1 mRNA Expression in Tendon Cells: An In Vitro Study. Connect Tissue Res.

[9] Sun HB, Andarawis-Puri N, Li Y, Fung DT, Lee JY, Wang VM, Basta-Pljakic J, Leong DJ, Sereysky JB, Ros SJ, Klug R a, Braman J, Schaffler MB, Jepsen KJ, Flatow EL (2010). Cycle-dependent matrix remodeling gene expression response in fatigue-loaded rat patellar tendons. J Orthop Res.

[10] Heinemeier K, Langberg H, Olesen JL, Kjaer M (2003). Role of TGF-beta1 in relation to exercise-induced type I collagen synthesis in human tendinous tissue. J Appl Physiol.

[11] Magnusson SP, Langberg H, Kjaer M (2010). The pathogenesis of tendinopathy: balancing the response to loading. Nat Rev Rheumatol.

[12] Langberg H, Skovgaard D, Petersen LJ, Bulow J, Kjaer M (1999). Type I collagen synthesis and degradation in peritendinous tissue after exercise determined by microdialysis in humans. J Physiol.

[13] Robling AG, Burr DB, Turner CH (2001). Recovery periods restore mechanosensitivity to dynamically loaded bone. J Exp Biol.

[14] Srinivasan S, Weimer D, Agans SC, Bain SD, Gross TS (2002). Low-magnitude mechanical loading becomes osteogenic when rest is inserted between each load cycle. J Bone Miner Res.

[15] Srinivasan S (2003). Enabling bone formation in the aged skeleton via rest-inserted mechanical loading. Bone.

[16] LaMothe JM, Zernicke RF (2004). Rest insertion combined with high-frequency loading enhances osteogenesis. J Appl Physiol.

[17] Hanson AD, Marvel SW, Bernacki SH, Banes AJ, van Aalst J, Loboa EG (2009). Osteogenic effects of rest inserted and continuous cyclic tensile strain on hASC lines with disparate osteodifferentiation capabilities. Ann Biomed Eng.

[18] Wang JH-C, Jia F, Gilbert TW, Woo SL-Y (2003). Cell orientation determines the alignment of cell-produced collagenous matrix. J Biomech.

[19] McBeath R, Pirone DM, Nelson CM, Bhadriraju K, Chen CS (2004). Cell shape, cytoskeletal tension, and RhoA regulate stem cell lineage commitment. Dev Cell.

[20] Li F, Li B, Wang QM, Wang JH (2008). Cell shape regulates collagen type I expression in human tendon fibroblasts. Cell Motil Cytoskeleton.

[21] Chuen FS, Chuk CY, Ping WY, Nar WW, Kim HL, Ming CK (2004). Immunohistochemical characterization of cells in adult human patellar tendons. J Histochem Cytochem.

[22] Maeda T, Sakabe T, Sunaga A, Sakai K, Rivera AL, Keene DR, Sasaki T, Stavnezer E, Iannotti J, Schweitzer R, Ilic D, Baskaran H, Sakai T (2011). Conversion of mechanical force into TGF-β-mediated biochemical signals. Curr Biol.

[23] Léjard V, Brideau G, Blais F, Salingcarnboriboon R, Wagner G, Roehrl MH a, Noda M, Duprez D, Houillier P, Rossert J (2007). Scleraxis and NFATc regulate the expression of the pro-alpha1(I) collagen gene in tendon fibroblasts. J Biol Chem.

[24] Sharma P, Maffulli N (2006). Biology of tendon injury: healing, modeling and remodeling. J Musculoskelet Neuronal Interact.

[25] Kongsgaard M, Kovanen V, Aagaard P, Doessing S, Hansen P, Laursen a H, Kaldau NC, Kjaer M, Magnusson SP (2009). Corticosteroid injections, eccentric decline squat training and heavy slow resistance training in patellar tendinopathy. Scand J Med Sci Sports.

[26] Wall ME, Weinhold PS, Siu T, Brown TD, Banes AJ (2007). Comparison of cellular strain with applied substrate strain in vitro. J Biomech.

[27] Stats Design & Analysis Web Guide. [http://dawg.utk.edu/diag/interpret_transformed_means.htm]

[28] Lavagnino M, Arnoczky SP, Kepich E, Caballero O, Haut RC (2008). A finite element model predicts the mechanotransduction response of tendon cells to cyclic tensile loading. Biomech Model Mechanobiol.

[29] Wall ME, Banes AJ (2005). Early responses to mechanical load in tendon: role for calcium signaling, gap junctions and intercellular communication. J Musculoskelet Neuronal Interact.

[30] Banes AJ, Weinhold P, Yang X (1999). Gap Junctions Regulate Responses of Tendon Cells Ex Vivo to Mechanical Loading. Clin Orthop Relat Res.

[31] McMahon GE, Morse CI, Burden A, Winwood K, Onambélé-Pearson GL (2013). The manipulation of strain, when stress is controlled, modulates in vivo tendon mechanical properties but not systemic TGF-β1 levels. Physiol Rep.

[32] Screen HRC, Shelton JC, Bader DL, Lee DA (2005). Cyclic tensile strain upregulates collagen synthesis in isolated tendon fascicles. Biochem Biophys Res Commun.

[33] Robbins JR, Evanko SP, Vogel KG (1997). Mechanical loading and TGF-beta regulate proteoglycan synthesis in tendon. Arch Biochem Biophys.

[34] Maniotis AJ, Chen CS, Ingber DE (1997). Demonstration of mechanical connections between integrins, cytoskeletal filaments, and nucleoplasm that stabilize nuclear structure. Proc Natl Acad Sci U S A.

[35] Chicurel ME, Chen CS, Ingber DE (1998). Cellular control lies in the balance of forces. Curr Opin Cell Biol.

[36] Malek AM, Izumo S (1996). Mechanism of endothelial cell shape change and cytoskeletal remodeling in response to fluid shear stress. J Cell Sci.

[37] Ippolito E, Natali PG, Postacchini F, Accinni L, De Martino C (1980). Morphological, Immunochemical and Biochemical Study of Rabbit Achilles Tendon at Various AGes. J Bone Joint Surg Am.

[38] Abraham T, Fong G, Scott A (2011). Second harmonic generation analysis of early Achilles tendinosis in response to in vivo mechanical loading. BMC Musculoskelet Disord.

[39] Wang JH-C (2006). Mechanobiology of tendon. J Biomech.

[CR40] The pre-publication history for this paper can be accessed here:http://www.biomedcentral.com/1471-2474/15/386/prepub

