# Supplementary figures and images for: Enhanced collagen type I synthesis by human tenocytes subjected to periodic in vitro mechanical stimulation
Source: BMC Musculoskelet Disord. 2014 Nov 21;15:386. doi: 10.1186/1471-2474-15-386 (PMC4256895; doi:10.1186/1471-2474-15-386)

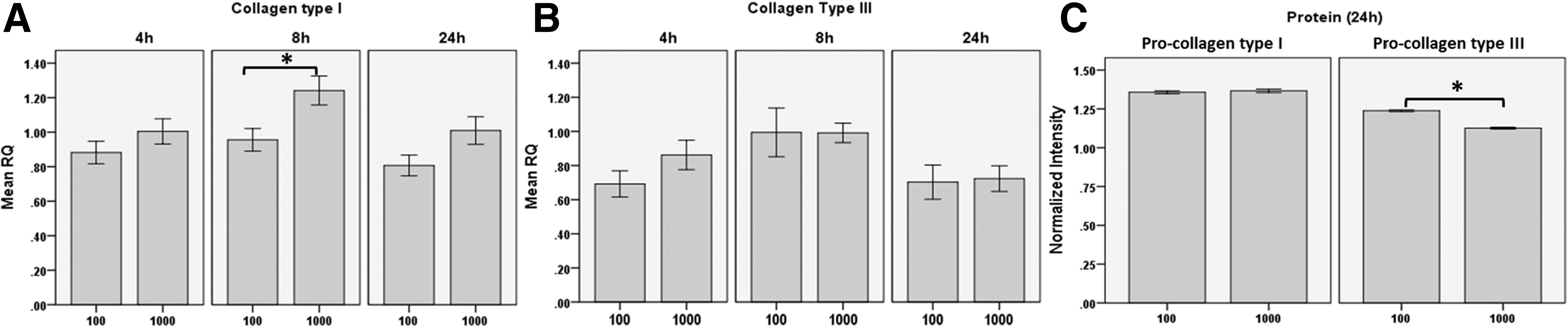

Supplement: Supplementary file 2 — Authors’ original file for figure 2 [file 12891_2014_2333_MOESM2_ESM.tif]

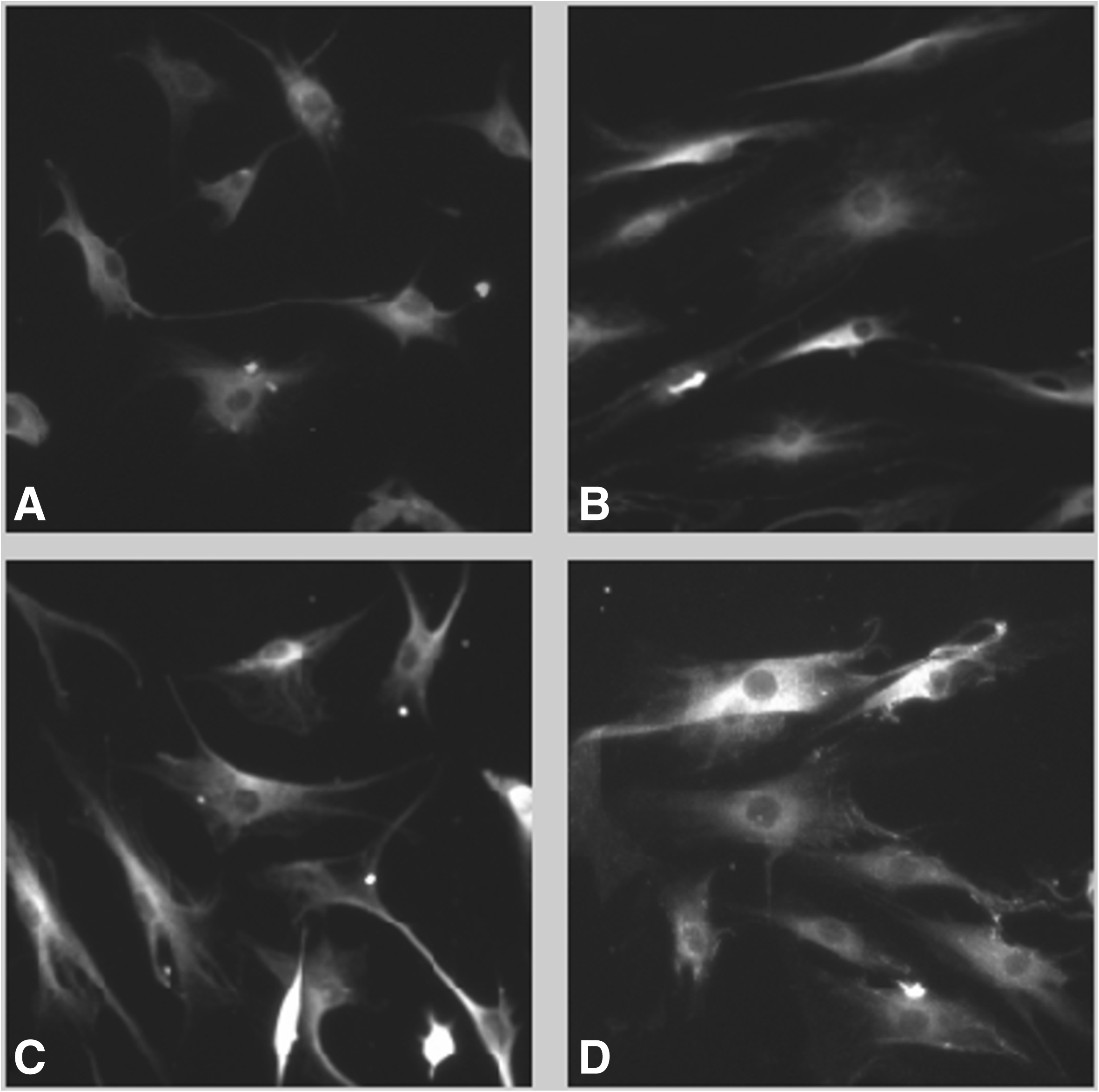

Supplement: Supplementary file 3 — Authors’ original file for figure 3 [file 12891_2014_2333_MOESM3_ESM.tif]

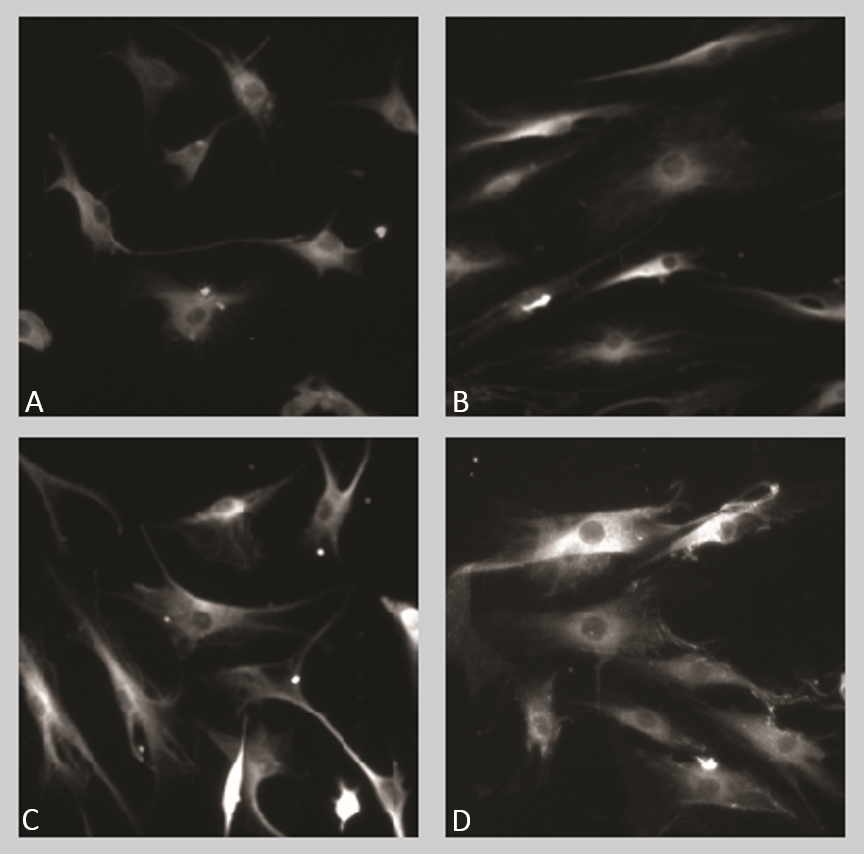

Supplement: Supplementary file 4 — Authors’ original file for figure 4 [file 12891_2014_2333_MOESM4_ESM.tiff]
